# Anatomical basis of the support of fibula to tibial plateau and its clinical significance

**DOI:** 10.1186/s13018-021-02500-8

**Published:** 2021-05-29

**Authors:** Wen-Bin Jiang, Shi-Zhu Sun, Chan Li, Philip Adds, Wei Tang, Wei Chen, Sheng-Bo Yu, Hong-Jin Sui

**Affiliations:** 1 Department of Anatomy, College of Basic Medicine, Dalian Medical University, Dalian, 116044 China; 2 Institute of Medical and Biomedical Education (Anatomy), St George’s, University of London, London, UK; 3 Department of Orthopedic Surgery, The Third Hospital of Hebei Medical University, Heibei, Shijiazhuang, 050051 China

**Keywords:** Tibia plateau, Fibula, Trabecular bone, Partial fibulectomy, Knee osteoarthritis

## Abstract

**Background:**

The fibula is only indirectly involved in the composition of the human knee joint and has therefore been neglected in the research on knee osteoarthritis. Nonuniform settlement of the proximal tibia plateau is clinically defined as when the height of the medial tibial plateau is lower than that of the lateral side in medial compartment knee osteoarthritis (KOA). The non-uniform settlement of the proximal tibia plateau may be caused by fibular support on the lateral side. Orthopedic surgeons practice partial fibulectomy based on the clinical manifestation of nonuniform settlement, and this technique has been shown to reduce pain and improve function in patients with medial compartment KOA. However, this hypothesis of the mechanism of nonuniform settlement lacks an anatomical basis.

**Methods:**

The P45 polyester plastination technique was used to prepare sections of the proximal tibiofibular joint to investigate the distribution of the bone trabeculae in the region of the lateral tibial plateau.

**Results:**

There was uneven distribution of trabeculae in the lateral condyle of the tibia and the head and neck of the fibula. The fibula and the posterolateral cortex of the shaft of the tibia united to form an arch beam via the tibiofibular joint. Many thick, dense trabeculae were present in a longitudinal direction above the tibiofibular arch.

**Conclusions:**

The fibula supports the lateral tibial plateau, and the trabeculae were concentrated above the tibiofibular arch.

## Introduction

Knee osteoarthritis (KOA), a degenerative joint condition, is one of the most prevalent chronic health conditions in the world, and is characterized by synovial membrane inflammation, subchondral bone sclerosis, articular cartilage degradation, and osteophyte formation [1]. The medial compartment of the knee is most commonly affected in those with osteoarthritis, and often leads to varus knee deformity. Identifying the risk factors and their pathogenesis has been a research hotspot in the field of joint surgery in recent years [2, 3]. Wang et al. [4] have argued that the changes in the proximal tibial plateau were evidenced in the dynamic changes of the tibia and fibula. They found that the level of the medial tibial plateau was lower than that of the lateral plateau in medial compartment KOA. The difference in height between these two positions has been defined as the “nonuniform settlement” of the proximal tibia plateau [5]. The reason for the lack of uniformity was presumed to be fibular support on the posterolateral wall of the tibial plateau. The construction of the tibial plateau would be inclined to accelerate this uneven phenomenon [3, 5], because it is mainly composed of cancellous bone. The slope of the tibial plateau caused by nonuniform settlement results in a transverse shearing force, with the femoral condyle producing ramping during walking and sports activities [6]. Studies on KOA have shown changes in congruity and redistribution of loading associated with radial displacement of the medial meniscus [7–9], and medial translation of the femoral condyles, seen on plain film X-rays of KOA patients, a phenomenon known as coronal tibiofemoral subluxation [10]. However, the pathogenesis of nonuniform settlement is not yet clear [11, 12]. It has been universally acknowledged that total knee arthroplasty (TKA) is the gold standard surgery for end-stage degenerative osteoarthritis. For OA limited to the medial compartment of the knee, high tibial osteotomy (HTO) or unicompartmental arthroplasty (UKA) may be used to avoid or postpone TKA [5]. Given the risks and high cost inherent with those surgeries, orthopedic surgeons in China have carried out partial fibulectomy, based on the manifestation of nonuniform settlement, and this simple surgical technique has been shown to be effective in reducing pain and improving function in patients with medial compartment OA [4]. However, the anatomical rationale of this theory has not yet been reported. In the present study, the P45 plastinated section technique was used to investigate the distribution of trabecular bone of the tibial-fibular region, to provide anatomical clues to elucidate the mechanism of nonuniform settlement of the tibial plateau, and the strategy of treating KOA.

## Materials and methods

### Materials

A total of 27 formalin-fixed adult knee joint specimens were studied. The specimens were taken from cadavers used in the teaching of human body dissection at Dalian Medical University. There were no signs of tumors, congenital malformations, fractures, severe osteoarthritis, or other related pathologies in the specimens selected for study. The Dalian Hoffen Biotechnology Co., Ltd., prepared serial sagittal, coronal, and horizontal sections of knee joints, using the P45 plastination technique, producing 15 sets of plastinated sagittal sections, 8 sets of plastinated coronal sections, and 4 sets of plastinated horizontal sections.

### Method

P45 plastination was carried out according to the standard procedure described by Sui and Henry [13]. Freeze that knee joint, and slicing (sliced with a high-speed band saw with a thickness of 3 mm), bleaching (after rinsing in cold water, soak overnight in 5% hydrogen peroxide), dehydration (100% acetone by the freeze-substitution process), vacuum impregnation (the sections was filled with Hoffen polyester P45, after, remove bubbles from the sections under vacuum) and curing (the sections were placed upright in a water bath at 40 °C for 3 days) proceeded sequentially. The detailed procedures refer to a previous study [14]. This study reported here focused on observing the relationship between the trabecular distribution of cancellous bone in the lateral tibial condyle and fibula.

## Result

### Oblique sagittal section through the proximal tibiofibular joint (Fig. 1)

**Fig. 1:**
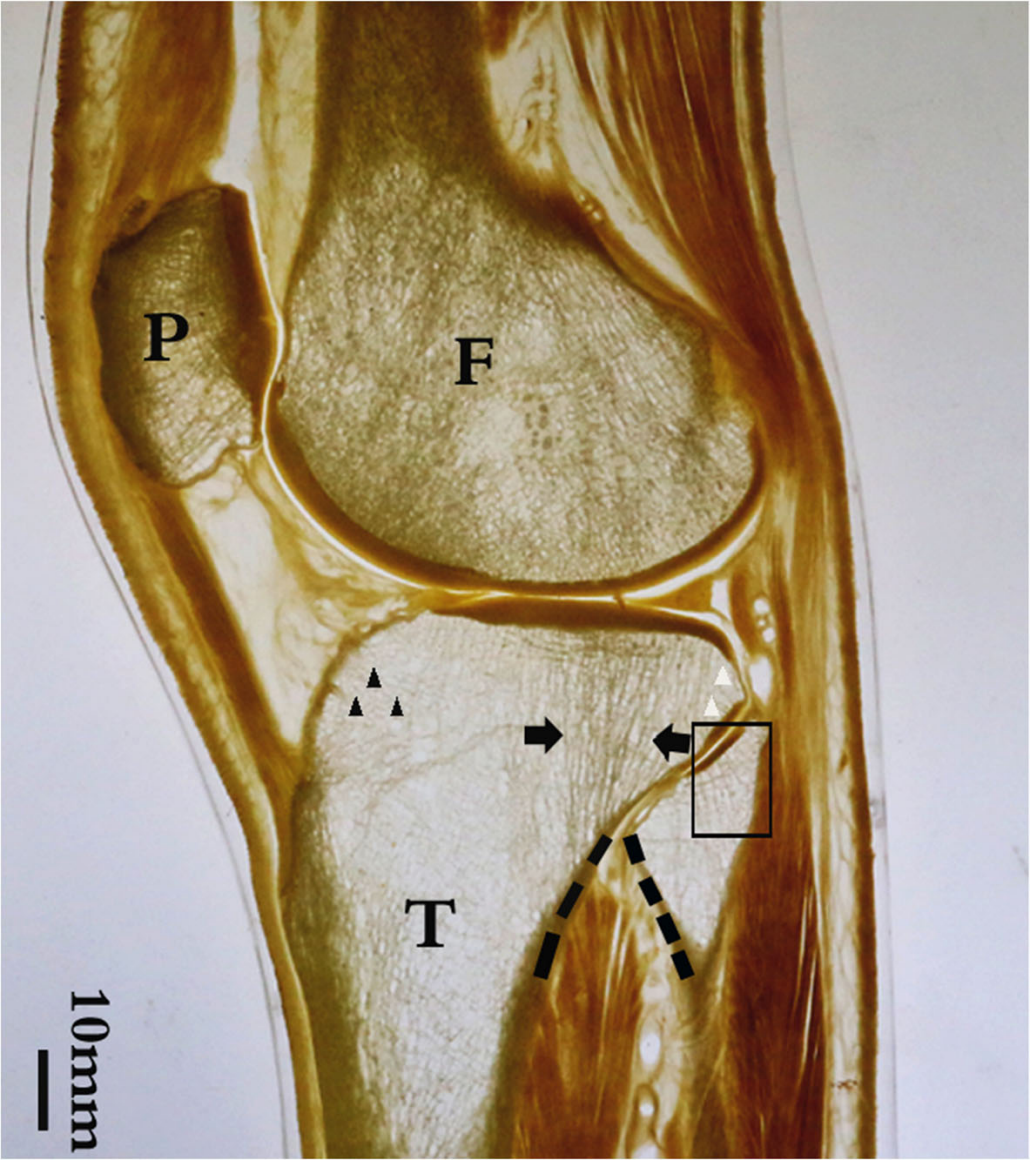
Parasagittal P45 section of the knee joint including the tibiofibular joint. The tiny and sparse bone trabeculae can be seen perpendicular to the tibiofibular joint surface, above the tibiofibular joint. The fibula forms an arch with the posterolateral cortex of the tibial shaft, through the tibiofibular joint. The distribution of trabecular bone in the lateral condyle of the tibia is nonuniform, while longitudinal trabeculae are densely distributed in the posterolateral part of the lateral tibial condyle. The longitudinal trabeculae passing through the epiphyseal line are distributed between the arch and the articular surface of the lateral tibial plateau. The posterior part of the fibular head shows parallel trabeculae perpendicular to the tibiofibular articular surface. In the anterior part of the fibular head, the internal trabeculae are sparse; no obvious longitudinal trabeculae are seen. F, femur; T, tibia; P, patella; dotted line, arch beam between the fibula and tibial plateau; black arrow, bone trabeculae arranged vertically. Black triangle, sparse trabeculae in anterior part of tibia. White triangle, sparse trabeculae in posterior tibia. Black square frame, longitudinally distributed trabeculae in the fibular head

**Fig. 2:**
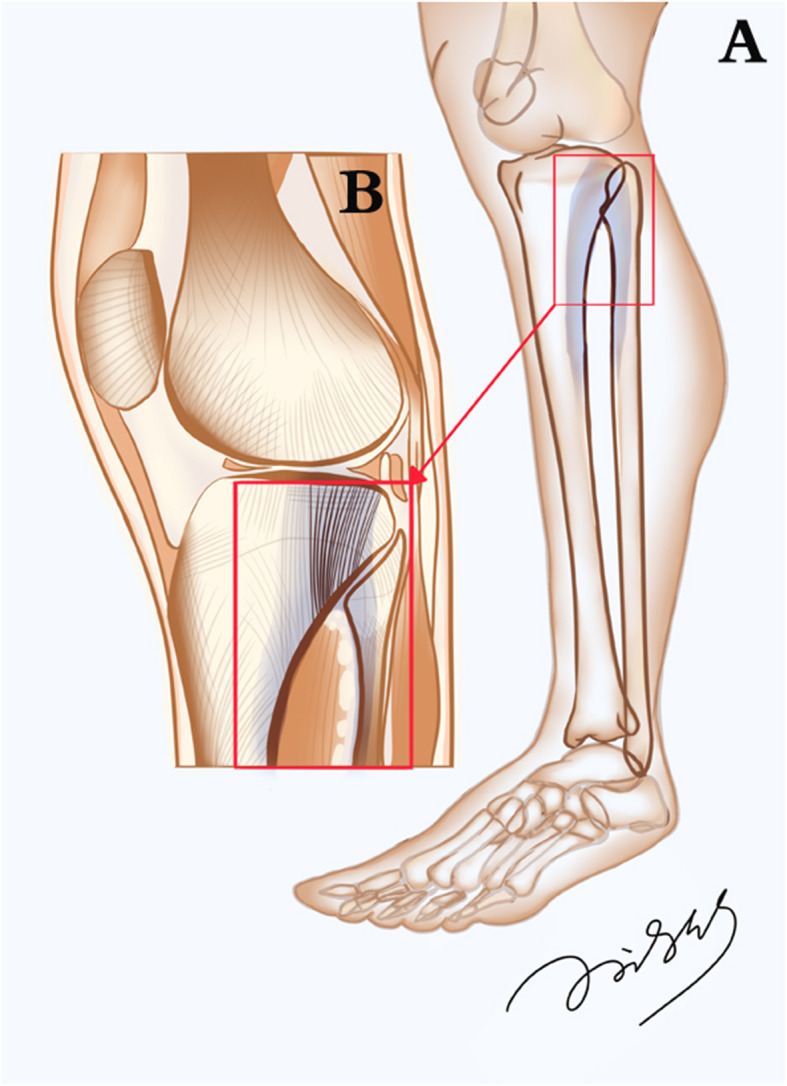
Schematic diagram of load-bearing arch beam composed of human tibia and fibula. (**A**) A load-bearing arch formed by the tibia and fibula. (**B**) Pattern diagram of Fig. 1. The tibiofibular arch might play an important role in the stress transfer of the lateral part of the tibial plateau

The lateral condyle of the femur and tibia and the lateral compartment of the knee joint were seen in these sections. The proximal tibiofibular joint, composed of the head of the fibula and the lateral condyle of the tibia, was showed on the posterolateral side of the tibial plateau. Uneven distribution of bone trabeculae was observed in the lateral condyle of the tibia. In the lateral condyle of the tibia, there were mainly longitudinal thick trabecular clusters beneath the articular surface (black arrow), while in the anterior part of the lateral condyle of the tibia, near the tibial tuberosity, the trabeculae were found to be sparse, very small, and reticulate (black triangle). In the posterolateral part of the lateral condyle of the tibia, the trabeculae above the tibiofibular joint were also small and sparse (white triangle). At the level of the metaphysis of the tibia, it was found that an arched construction was formed by the union of the shaft of the fibula and the posterior lateral bone cortex of the shaft of the tibia, via the tibiofibular joint (the dotted line in Figs. 1 and 2). The tibiofibular joint acted as the keystone of the arch. The longitudinal dense trabeculae passing through the epiphyseal line of the tibia were distributed between the vault of the arch and the articular surface of the lateral tibial plateau (black arrow). Furthermore, the trabeculae in the fibular head were uneven, which are the trabeculae of the posterolateral part of the fibular head (black square frame) were denser than those of the anterior medial part.

### Coronal section through the tibiofibular joint (Fig. 3)

The coronal sections showed the medial and lateral condyles of the femur, the posterior part of the tibial plateau, and the tibiofibular joint. The distribution of bone trabeculae in the tibial plateau was uneven. In the inferior projection area of the articular surface of the tibial plateau, the trabeculae were thick and dense. The trabeculae originated at the articular surface, penetrated the epiphyseal line, and then terminated at the sloping bone cortex of the posterior wall of the metaphysis of the tibia (black triangle). The bone trabeculae in the lateral condyle of the tibial plateau (black triangle) were slightly denser than those in the medial condyle (white triangle); the cortex was thickened accordingly on the lateral side of the oblique posterior wall of the tibial metaphysis. However, in the lateral edge of the lateral condyle, above the tibiofibular joint, the trabeculae were thin and irregular. Thin and sparse trabeculae were also present in the head of the fibula (black square frame).

**Fig. 3:**
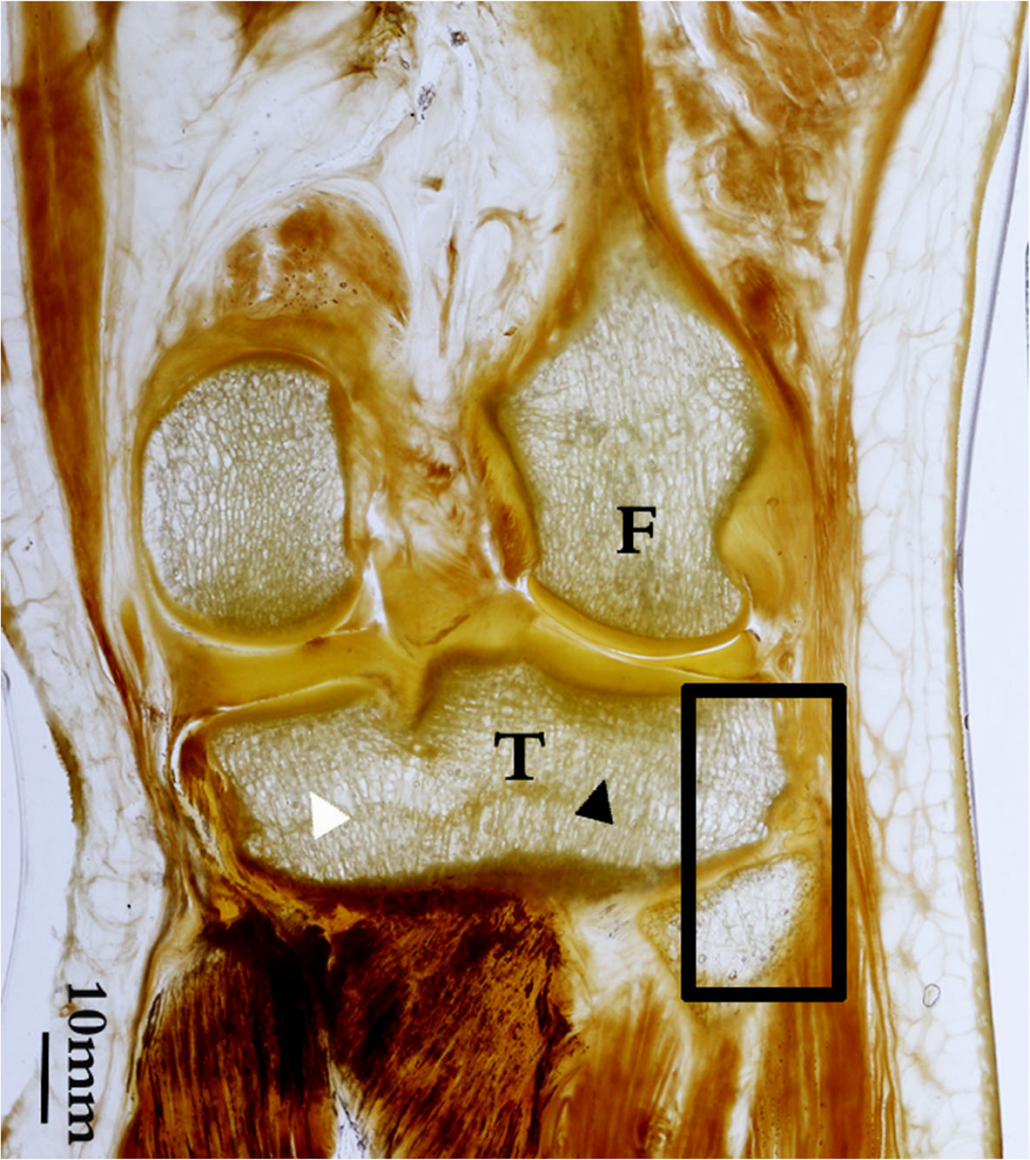
Coronal P45 section of the knee joint (through the tibiofibular joint**)**. In the lateral condyle of the tibia, the trabeculae above the fibular head are tiny and sparsely reticulated, accounting for about one-quarter of the width of the lateral condyle. The thicker, longitudinally arranged trabeculae can be seen between the articular surface and the epiphyseal bone cortex in the remaining three-quarters. There are sparse trabeculae in the fibular head. F, femur; T, tibia; black triangle, dense trabeculae in lateral condyle of tibial plateau. White triangle, dense trabeculae in medial condyle of tibial plateau. Black square frame, the bone trabeculae were irregular in this area of the lateral tibial plateau and fibula

### Horizontal section near the neck of the fibula (Fig. 4)

This section showed the structure of the metaphysis of the tibia and the head of the fibula. The metaphysis of the tibia was triangular in shape, and a large number of dense and reticulated trabeculae were found in three regions: near the posterior lateral cortex (a), the medial cortex (b), and deep to the tibial tuberosity (c). Similarly, dense trabeculae were formed in a reticulated pattern in the posteromedial half of the head of the fibula (d). These areas of dense and reticulated trabeculae in the metaphysis of the tibia and the head of the fibula were united by the tibiofibular joint, and could form a solid trabecular foundation of the knee, i.e., a tripartite construction of dense trabecular areas.

**Fig. 4:**
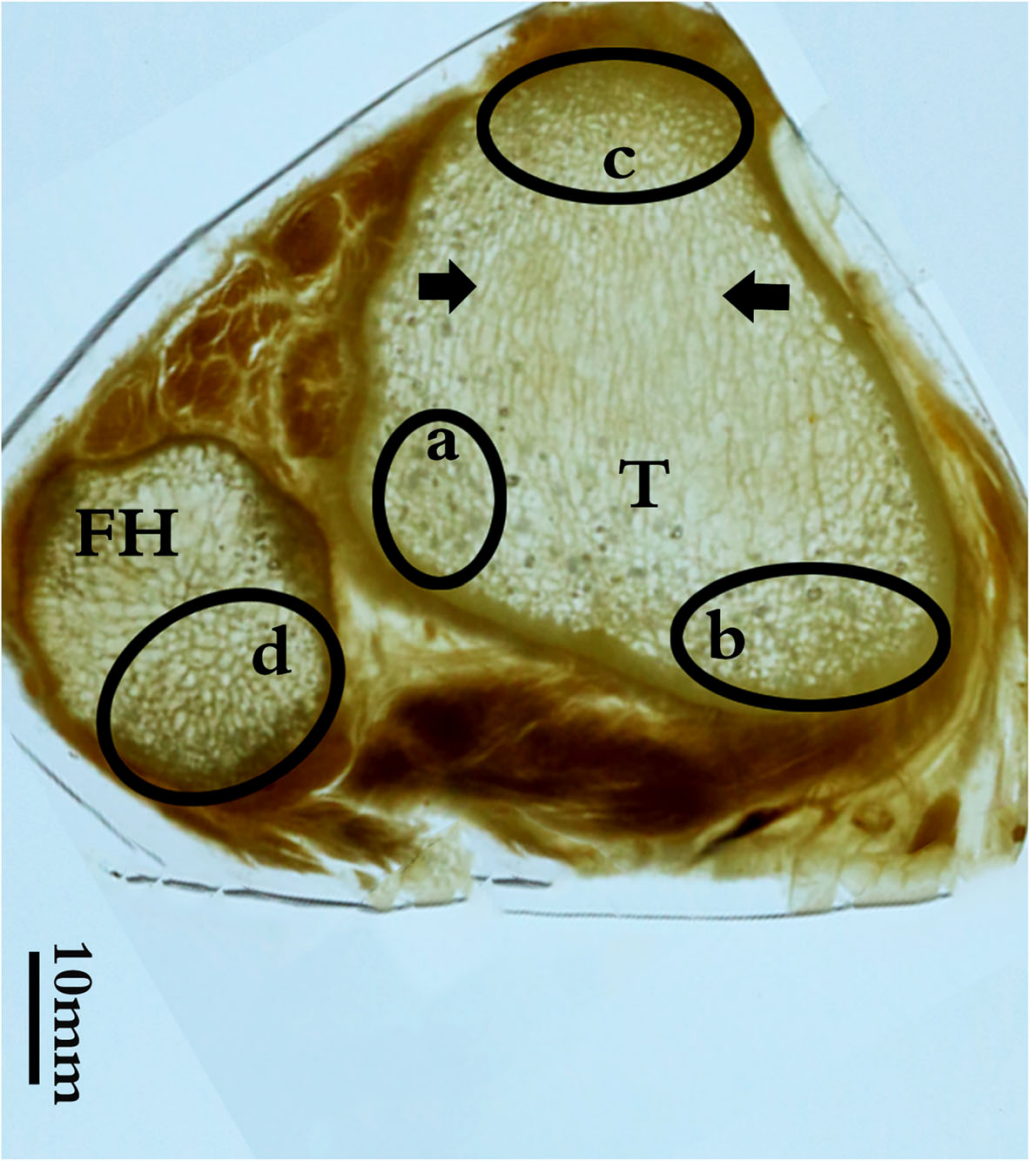
Axial P45 section of the knee joint through the tibiofibular joint. Many reticulated trabeculae are visible near the lateral (a) and posteromedial (b) tibial cortex. In the central area of the metaphysis of the tibia, the trabeculae are parallel, in a sagittal orientation (black arrows). There are numerous thickened trabeculae on the deep surface of the tibial tuberosity (c). Contrastingly, the cancellous bone of the fibula is mainly composed of grid-like trabeculae, mostly located in the posterior region of the fibular head (d). The dense, reticulated trabeculae in the metaphysis of the tibia and the posterior half area of the neck of the fibula may be united by the tibiofibular joint to form a tripartite construction. T, the metaphysics of the tibia; FH, fibular head; the black ellipse, the reticulated trabecular area densely distributed in the fibula and tibia

## Discussion

According to the currently held view, the fibula is not directly involved in the composition of the knee joint. The fibula, therefore, has been neglected in research on knee osteoarthritis [4]. In the current study, it was found that there was an uneven distribution of trabeculae in the lateral condyle of the tibia and the head and neck of the fibula. In the lateral condyle of the tibia, a large number of thick trabeculae were distributed longitudinally in clusters beneath the articular surface, which then penetrated the epiphyseal line and terminated on the slope of the posterior cortex of the metaphysis of the tibia. The cortical slope formed one side of a stable arch beam, constructed by the union of the fibula with the cortex of the posterolateral side of the metaphysis of the tibia, via the tibiofibular joint. This suggested that the tibiofibular arch could play an important role in bearing the downward forces of the lateral compartment of the knee joint. This was further supported by the observation that concentrated reticular trabeculae were present in the posteromedial area of the neck of the fibula, which could provide the internal strength for the bracing of the fibular head. As a result, the tibiofibular joint would form an indispensable fulcrum for the mechanical arch between the tibia and fibula. Furthermore, thin and sparse trabeculae were located above the tibiofibular joint, in the posterolateral marginal region of the tibial plateau, suggesting that the fibula might not directly bear the stress originating from the articular surface of the knee.

Previous studies have shown that the proximal tibia mainly comprises cancellous bone. The bone density of the proximal fibula and tibia decreases in the following order: fibula, medial plateau, lateral plateau, and middle proximal tibia [5]. McNeil et al. [15] have reported that, while there may be significant loss of cortical bone thickness in the proximal tibia with advancing age, there is likely to be little loss from the proximal fibula, suggesting that the fibula is not subject to age-related bone loss. It follows then, that the fibula will maintain its strength more than the tibial plateau. In the present study, it was observed that the mechanical arch beam was formed by the union of the fibula and the tibial plateau, and the tibiofibular joint formed the constant keystone of the arch on the posterolateral side of the tibial plateau. When moving or walking upright, gravity transmitted by the articular surface of the lateral condyle of the tibia would fall on to the top of the arch. The dense and reticulated trabeculae in the tibia and fibula might be combined with each other by the tibiofibular joint, to form a tripartite construction. Consequently, the fibula may play a key role in transmitting the weight, and kinetic energy in motion, of the posterolateral aspect of the knee.

The process of human aging is accompanied by a decrease in bone mass, especially in the load-bearing joints such as the knees, hips, ankles, and spine [16]. A supporting and bracing effect on the lateral tibial plateau is supplied through the proximal tibiofibular joint by the strong fibula. This could also be the anatomical and pathological mechanism of nonuniform settlement of the tibial plateau. It has been indirectly indicated that the mechanical load on the medial and lateral tibial condyles are unevenly distributed [17–20]. There has also been evidence that the progression of KOA may be related to the distribution of tibiofemoral articular stress [21]. Studies have found that the sloping of the tibial plateau arising from nonuniform settlement results in a transverse shearing force, with the femoral condyle shifting medially during walking and sports [6]. These observations should bring the clinician’s attention to the role of the fibula in the coronal subluxation of the proximal tibia in patients with knee osteoarthritis.

Moreover, the geometry of the tibial plateau has attracted more and more attention from researchers in recent years. A study has shown that the inclination of the tibial plateau may be a predictive risk factor for anterio cruciate ligament injury [22]. According to the results of the present study, the fibula may affect the inclination of the tibial plateau, especially in osteoarthritis patients with varus knees. Therefore, for medial KOA, the role of the fibula in supporting the lateral tibial plateau should be of considerable interest to clinicians.

Collectively, the findings in the present study have revealed a novel correlation between the proximal fibula and tibia, which might provide an anatomical basis for the further treatment of KOA, and an understanding of the risk factors for anterio cruciate ligament injury.

### Limitations

In future research, quantifying the information of trabecular bone at the arched beam will have important implications for further treatment of KOA.

## Conclusion

An arch beam was formed by the union of the fibula and the posterolateral cortex of the tibial shaft via the proximal tibiofibular joint. This may play an important role in the transfer of mechanical loads from the articular surface of the lateral condyle of the tibia. The tibiofibular joint forms an indispensable keystone for the arch beam. The fibula may be an important factor in the diagnosis and treatment of knee pathology.

## Data Availability

The datasets used and/or analyzed during the current study are available from the corresponding author on reasonable request.

## Abbreviations

KOA: Knee osteoarthritis
TKA: Total knee arthroplasty
